# Fundamental Insights into Copper-Epoxy Interfaces for High-Frequency Chip-to-Chip Interconnects

**DOI:** 10.1021/acsami.4c16414

**Published:** 2024-12-18

**Authors:** Junghyun Park, Monsuru Dauda, Mustapha Bello, Ignace Agbadan, Anthony Christian Engler, Jaimal M. Williamson, Varughese Mathew, Sunggook Park, John C. Flake

**Affiliations:** † Gordon A. and Mary Cain Department of Chemical Engineering, Louisiana State University, Baton Rouge, Louisiana 70803, United States; ‡ Texas Instruments Incorporated, Dallas, Texas 75243, United States; § NXP Semiconductors, Austin, Texas 78735, United States; ∥ Department of Mechanical & Industrial Engineering and Center for Bio-Modular Multiscale Systems, Louisiana State University, Baton Rouge, Louisiana 70803, United States

**Keywords:** multichip package, chip-to-chip (C2C) interconnect, surface modification, oxidation, silane coupling agent, dipodal amine functional silane, polymer−metal adhesion

## Abstract

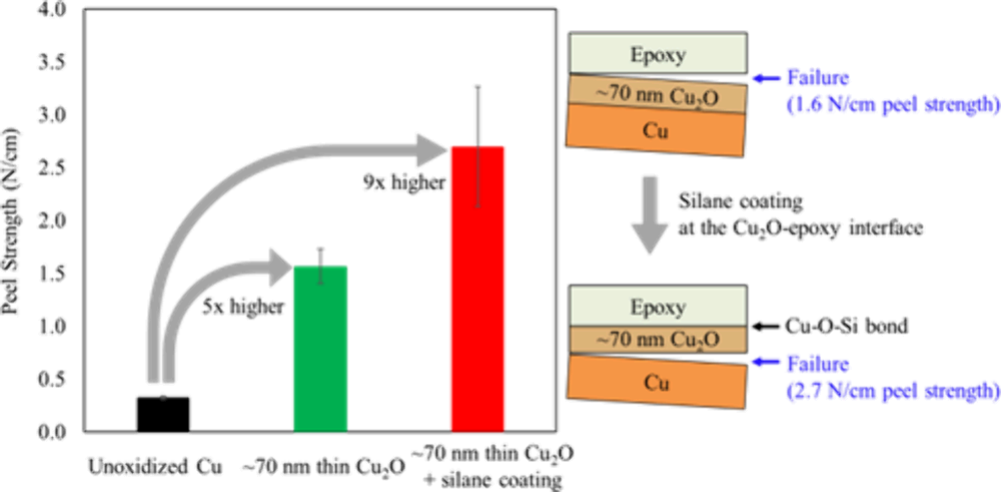

Future processes and materials are needed to enable multichip packages with chip-to-chip (C2C) data rates of 50 GB/s or higher. This presents a fundamental challenge because of the skin effect, which exacerbates signal transmission losses at high frequencies. Our results indicate that smooth copper interconnects with relatively thin cuprous oxides (Cu_2_O, Cu^I^) and amine-functional silane adhesion promoters improve interfacial adhesion with epoxy dielectrics by nearly an order of magnitude. For the first time, we present X-ray photoelectron spectroscopy (XPS) and Raman spectroscopy evidence of Cu(I)–O–Si bond formation at silane-treated interfaces. Thus, relatively smooth interconnects can benefit from reduced skin losses while maintaining their mechanical integrity and reliability. Failure mechanisms of Cu interconnects with cuprous and cupric oxide (CuO, Cu^II^) are explored using scanning electron microscopy (SEM) and Auger electron spectroscopy (AES). These results indicate that both cupric oxides and relatively thick cuprous oxide interfaces lead to relatively weaker interfaces compared with thin cuprous oxides with adhesion promoters.

## 1 Introduction

Advanced multichip packages with high-performance chip-to-chip (C2C) interconnects are needed for new applications in artificial intelligence (AI),^1−3^ virtual reality,^3−5^ 6G communication,^6−8^ and autonomous vehicles.^9−11^ As recently announced at the 2024 GPU Technology Conferences, new multichip architectures are expected to enable up to 1.8 TB/s per GPU or 50 GB/s per C2C interconnect for trillion-parameter AI models.^12^ While low-loss optical interconnects have been proposed for relatively long-distance interconnects, the additional costs and latency associated with electrical-optical-electrical signal conversion pose barriers to their adoption for conventional multichip packages and C2C transmission distances.^13,14^ Thus, there is significant interest in understanding the limits of copper interconnections with polymer dielectrics.

In one approach for C2C interconnects at high-bandwidth applications, Mahajan et al. proposed embedded multidie interconnect bridges (EMIB) using copper (Cu) interconnects with epoxy dielectrics.^1,15^ This 2.5D approach extends the use of conventional packaging materials with well-known performance and reliability metrics; however, several challenges exist in extending these types of interconnects to GHz frequencies. Recent studies have reported relatively high insertion losses of 0.8 dB up to 200 GHz^16^ and 1.5 dB up to 300 GHz.^17,18^ These high insertion losses are associated with inductance from electromagnetic interference (EMI) and the skin effect, where the current concentrates near the conductor’s surface.^19^

Interconnects with smooth interfaces are more desirable for high-frequency applications because roughness tends to exacerbate signal losses due to the skin effect.^20,21^ Conversely, mechanical reliability metrics such as adhesion are typically compromised with smoother interfaces.^22−24^ Thus, there is a growing need to build a better understanding of the interfacial chemistry between Cu and polymer dielectrics. More broadly, Cu–polymer interfaces are commonly found in most microelectronics devices;^25,26^ however, there have only been a few studies that explicitly consider their performance or reliability at high frequencies.^25^

Previous works have shown that adhesion at Cu-epoxy interfaces may be improved by surface treatments such as wet-etching,^26,27^ oxidation,^28,29^ and UV ozone treatments.^30,31^ Roughening via oxidation is commonly employed due to its low cost and simplicity.^29^ Previous works indicated that a rough cupric oxide (CuO, Cu^II^) layer improves mechanical interlocking due to its needle-like morphology.^28^ In general, polymer adhesion to the metal depends on the thickness of Cu oxides.^29^ Relatively thin (<70 nm) Cu oxides were considered to increase adhesion strength,^32^ and relatively thick oxides (>150 nm) led to delamination failures.^33,34^ The previous works also suggested that relatively thin Cu oxides were beneficial in terms of reliability; however, they did not explicitly consider the oxidation state or adhesion failure mechanisms.

In addition to oxidation, several practitioners use adhesion promoters such as silane coupling agents to improve the integrity of the Cu–polymer interface. As noted by Nambafu et al., dipodal silanes, which contain more branches with hydrolyzable silanol groups, have been shown to enhance hydrolytic stability;^35,36^ however, there was no evidence of covalent bonding.

This study explores the interfacial chemistry of Cu interconnects with epoxy dielectrics by using Cu oxides and an amine-functionalized dipodal silane adhesion promoter. Two oxidants, sodium persulfate (Na_2_S_2_O_8_) and hydrogen peroxide (H_2_O_2_), are used to produce cuprous oxide (Cu_2_O, Cu^I^) and cupric (CuO, Cu^II^) films, respectively. Oxide thickness is presented by scanning electron microscopy (SEM). The impact of Cu_2_O and CuO on the failure mechanism is investigated by Auger electron spectroscopy (AES) at different thicknesses. X-ray photoelectron spectroscopy (XPS) and Raman spectroscopy were used to evaluate the surface states and binding environments.

## 2 Experimental Section

### 2.1 Materials

0.01 mm-thick 99.9% Cu foil was obtained from MilliporeSigma, USA. Na_2_S_2_O_8_ was purchased from Thermo Scientific, USA. 30% H_2_O_2_ was obtained from Thermo Scientific, USA. 92% Bis(methyldiethoxysilylpropyl)amine (BMSPA) was purchased from Gelest, USA. Epoxy blends included diglycidyl ether of bisphenol A (DGEBA, EPON 828, Miller-Stephenson Chemicals, USA), methyl-5-norbornene-2,3-dicarboxylic anhydride (Nadic methyl anhydride, NMA, Electron Microscopy Sciences, USA), and imidazole (Sigma-Aldrich. USA).

### 2.2 Surface Oxidation

Cu foils were cleaned with 10% hydrochloric acid (HCl) for 10 min before surface oxidation. Na_2_S_2_O_8_ and H_2_O_2_ solutions were used to grow Cu_2_O and CuO, respectively.^28^ Cu foils were oxidized by either 0.1 M Na_2_S_2_O_8_ or 30% H_2_O_2_ for 10 or 60 min as shown in Table 1: the Na_2_S_2_O_8__10 min sample was oxidized by Na_2_S_2_O_8_ for 10 min, the Na_2_S_2_O_8__60 min sample was oxidized by Na_2_S_2_O_8_ for 60 min, the H_2_O_2__10 min sample was oxidized by H_2_O_2_ for 10 min, and the H_2_O_2__60 min sample was oxidized by H_2_O_2_ for 60 min. The surface morphology and thickness of the Cu oxides were measured by SEM (FEI Quanta 3D FIB-SEM, USA). The cross sections of the samples were prepared by cutting the center of the Cu foils using ethanol-cleaned scissors. Atomic force microscopy (Horiba SmartSPM AIST, Japan) quantified surface roughness after oxidation using an AFM tip with a radius ≤8 nm and a scan rate of 1.0 Hz in tapping mode. AFM measurements from 3 samples were collected to determine the error bars.

**Table 1:** Conditions of Oxidation and Silanization Applied on Cu Foils

Name	Oxidation	Silanization
Unoxidized Cu	Not oxidized	Not silanized
Unoxidized Cu+BMSPA	Not oxidized	3% BMSPA for 20 min
Na_2_S_2_O_8__10 min	0.1 M Na_2_S_2_O_8_ for 10 min	Not silanized
Na_2_S_2_O_8__10 min+BMSPA	0.1 M Na_2_S_2_O_8_ for 10 min	3% BMSPA for 20 min
Na_2_S_2_O_8__60 min	0.1 M Na_2_S_2_O_8_ for 60 min	Not silanized
Na_2_S_2_O_8__60 min+BMSPA	0.1 M Na_2_S_2_O_8_ for 60 min	3% BMSPA for 20 min
H_2_O_2__10 min	30% H_2_O_2_ for 10 min	Not silanized
H_2_O_2__10 min+BMSPA	30% H_2_O_2_ for 10 min	3% BMSPA for 20 min
H_2_O_2__60 min	30% H_2_O_2_ for 60 min	Not silanized
H_2_O_2__60 min+BMSPA	30% H_2_O_2_ for 60 min	3% BMSPA for 20 min

### 2.3 Silanization

Oxidized copper foils were dipped into a 3% BMSPA solution for 20 min. The BMSPA-coated samples are labeled with ‘+BMSPA,’ as presented in Table 1. The solvent of the BMSPA solution consisted of 10% deionized water and 90% ethanol. Silanized Cu oxides were rinsed with ethanol and heated at 80 °C for 15 min. Reinshaw inVia Reflex Raman Microscope (Reinshaw, UK) was used at an excitation wavelength of 532 nm to characterize chemical phases of the Cu oxide surface before and after BMSPA coating. The Raman spectra were collected with a 10 s exposure time. The baseline of the spectra was subtracted from the WiRE software.

### 2.4 Formation of Cu-Epoxy Joints

**Figure 1:**
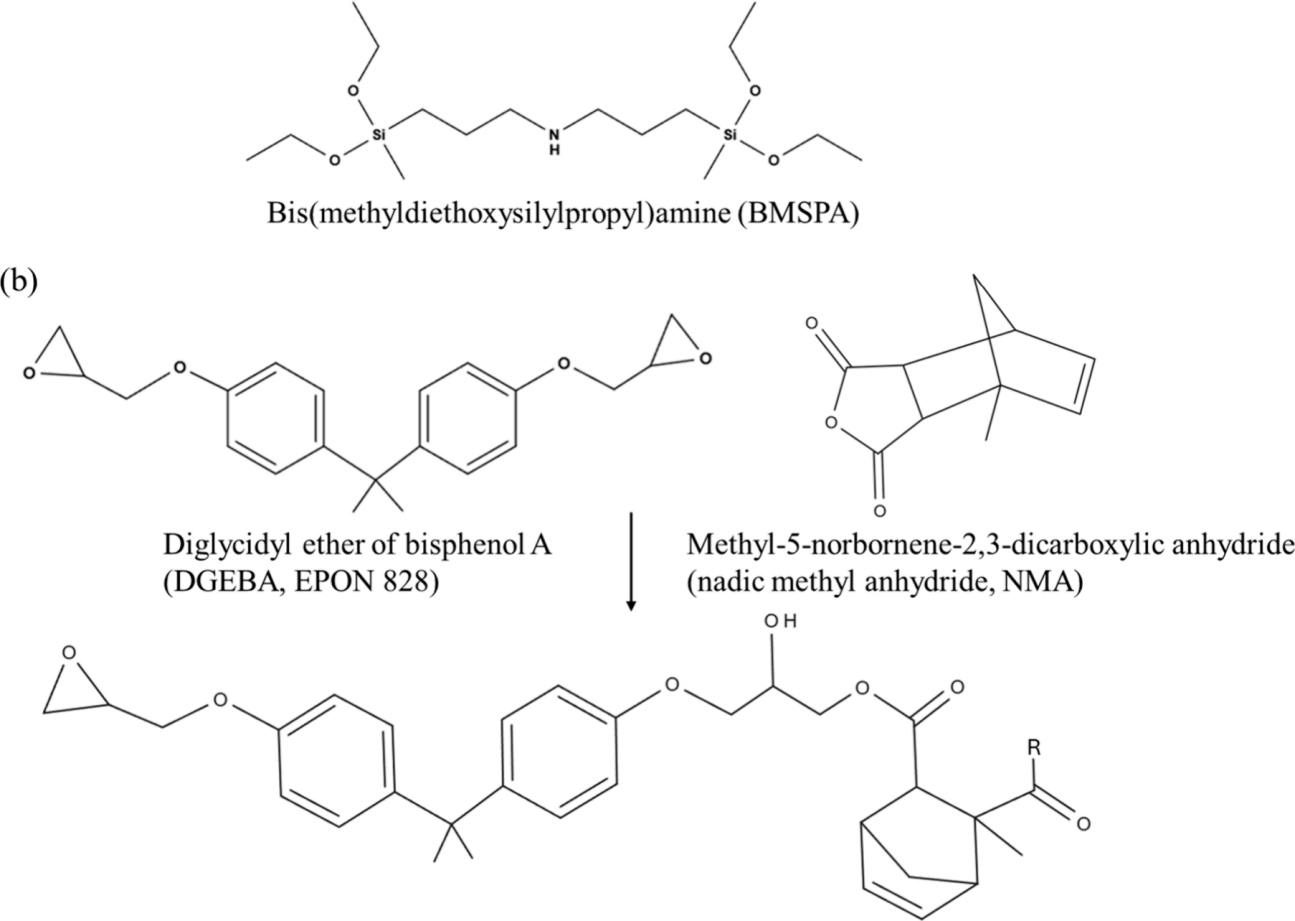
(a) Chemical structure of bis(methyldiethoxysilylpropyl)amine (BMSPA) and (b) curing reaction of diglycidyl ether of bisphenol A (DGEBA, EPON 828) and methyl-5-norbornene-2,3-dicarboxylic anhydride (nadic methyl anhydride, NMA).

The epoxy blend comprised 52% EPON 828, 47% NMA, and 1% imidazole. Epoxy was cured by pouring the mixture onto the silanized Cu oxides and heating for 8 h at 115 °C.^22,37^ During the curing process of epoxy on silane-coated Cu, the silane grafted to epoxy resin is reported to form a bond; the amine functional group of the silane in Figure 1a is reported to bond with the epoxide ring in the epoxy.^38,39^ The epoxide also participates in curing reaction with NMA by opening the epoxide ring.^22^Figure 1b shows that an isopropyl segment is formed between the DGEBA resin and NMA by opening the cyclic ether ring of epoxides during the curing process of the epoxy.^22^Figure S1 shows that the center of the Cu layer was cut by a sharp knife to prepare a 10 mm-wide Cu strip for a 90° peel test. The Cu strip was peeled off at a 25 mm/min rate according to ASTM B-533.^40^ AES or XPS (Scienta Omicron ESCA 2SR X-ray Photoelectron Spectroscope, Sweden) was carried out on (i) Cu surfaces before molding epoxy, (ii) Cu surfaces after the peel test of Cu-molded epoxy, and (iii) epoxy surfaces after the peel test of Cu-molded epoxy. The samples were stored in a vacuum chamber prior to XPS scans to prevent further oxidation. CasaXPS software was utilized to deconvolute the O 1s spectra peaks of silanized Cu using the Tougaard background.^41^ A monochromatic Al Kα (1486.7 eV) X-ray source was used at a power of 300 W (15 kV) with a pass energy of 30 eV, dwell time of 1 s, and 0.5 eV step.

## 3 Results and Discussion

### 3.1 Oxide Morphology

**Figure 2:**
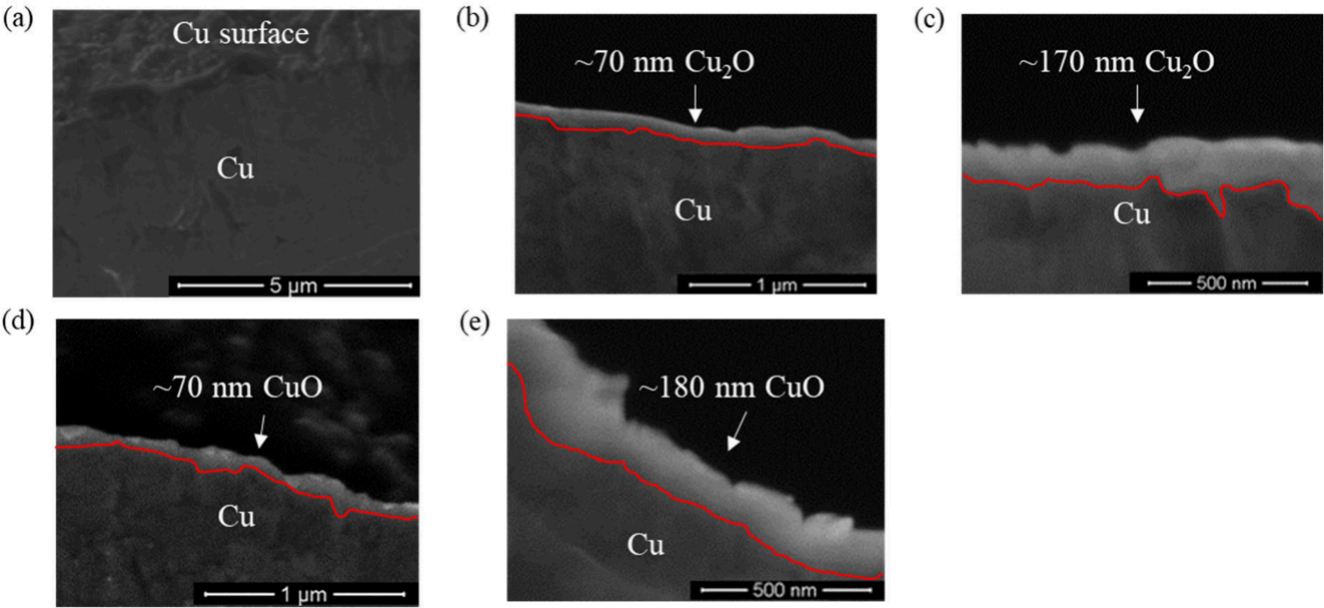
Cross-sectional SEM images of (a) nonoxidized Cu foil, Cu foils oxidized (b) by Na_2_S_2_O_8_ for 10 min, (c) by Na_2_S_2_O_8_ for 60 min, (d) by H_2_O_2_ for 10 min, and (e) by H_2_O_2_ for 60 min. The darker and brighter regions correspond to Cu and Cu oxide, respectively. Red lines indicate the interface between Cu and the Cu oxides.

**Figure 3:**
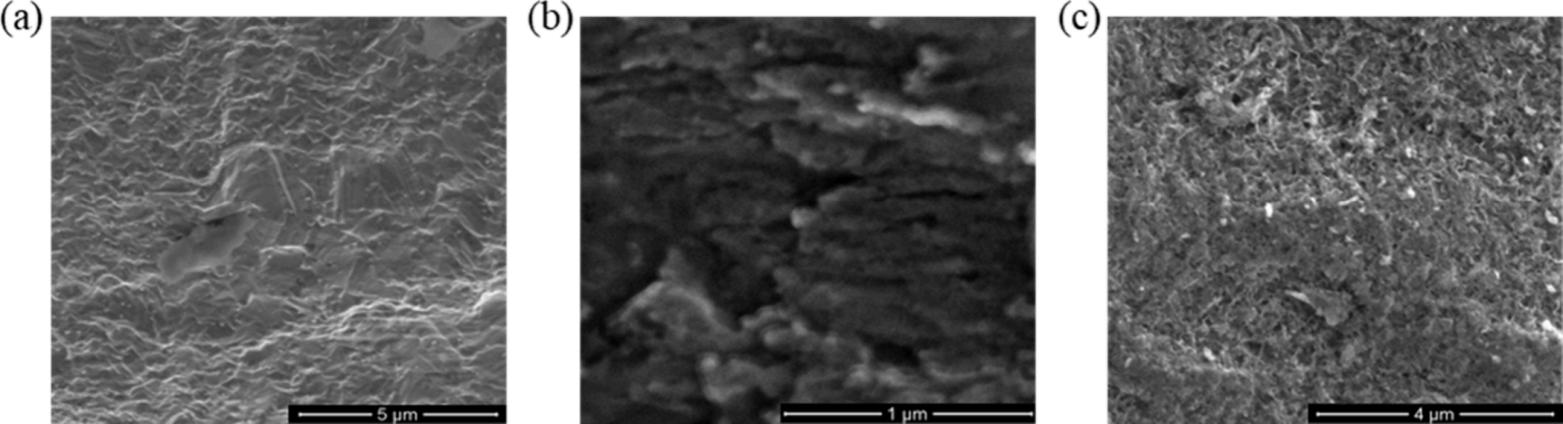
Surface SEM images of (a) nonoxidized Cu foil, (b) Cu foil oxidized by Na_2_S_2_O_8_ for 10 min, and (c) Cu foil oxidized by H_2_O_2_ for 10 min.

SEM images in Figure 2a-e present cross-sectional areas. SEM images in Figure 3a-c present the surface morphology of the oxidized Cu foils. In Figure 2a-e, the darker and brighter regions correspond to the Cu and Cu oxide, respectively, which is supported by the EDS-SEM results in Figures S2 and S3.^32^ Oxidation with Na_2_S_2_O_8_ increases the thickness of Cu oxide to ∼70 nm after 10 min and to ∼170 nm after 60 min. Treatment with peroxide, H_2_O_2_, results in an ∼70 nm Cu oxide layer after 10 min and an ∼180 nm layer after 60 min. Figure 3a shows that the nonoxidized Cu foil has a relatively smooth surface. In contrast, Figure 3b presents that the oxidation of Cu with Na_2_S_2_O_8_ forms uneven and flake-like structures.^28^ The H_2_O_2_ treatment is reported to form CuO,^28^ having a monoclinic crystal structure.^29,42^Figure 3c shows that this monoclinic structure promotes the formation of needle-like particles.^43,44^

**Figure 4:**
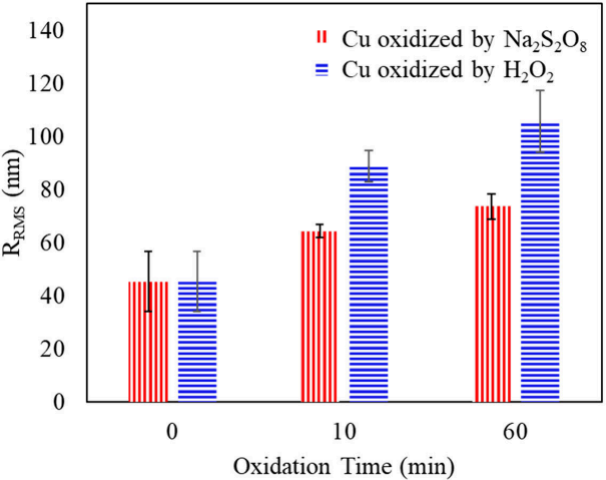
R_RMS_ of the Cu surface oxidized by Na_2_S_2_O_8_ or H_2_O_2_ as a function of oxidation time. Error bars are determined by measuring three samples. Data and error limits for the samples are as follows: Unoxidized Cu: 45 ± 11 nm, Na_2_S_2_O_8__10 min: 64 ± 3 nm, Na_2_S_2_O_8__60 min: 74 ± 5 nm, H_2_O_2__10 min: 89 ± 6 nm, H_2_O_2__60 min: 106 ± 12 nm.

**Figure 5:**
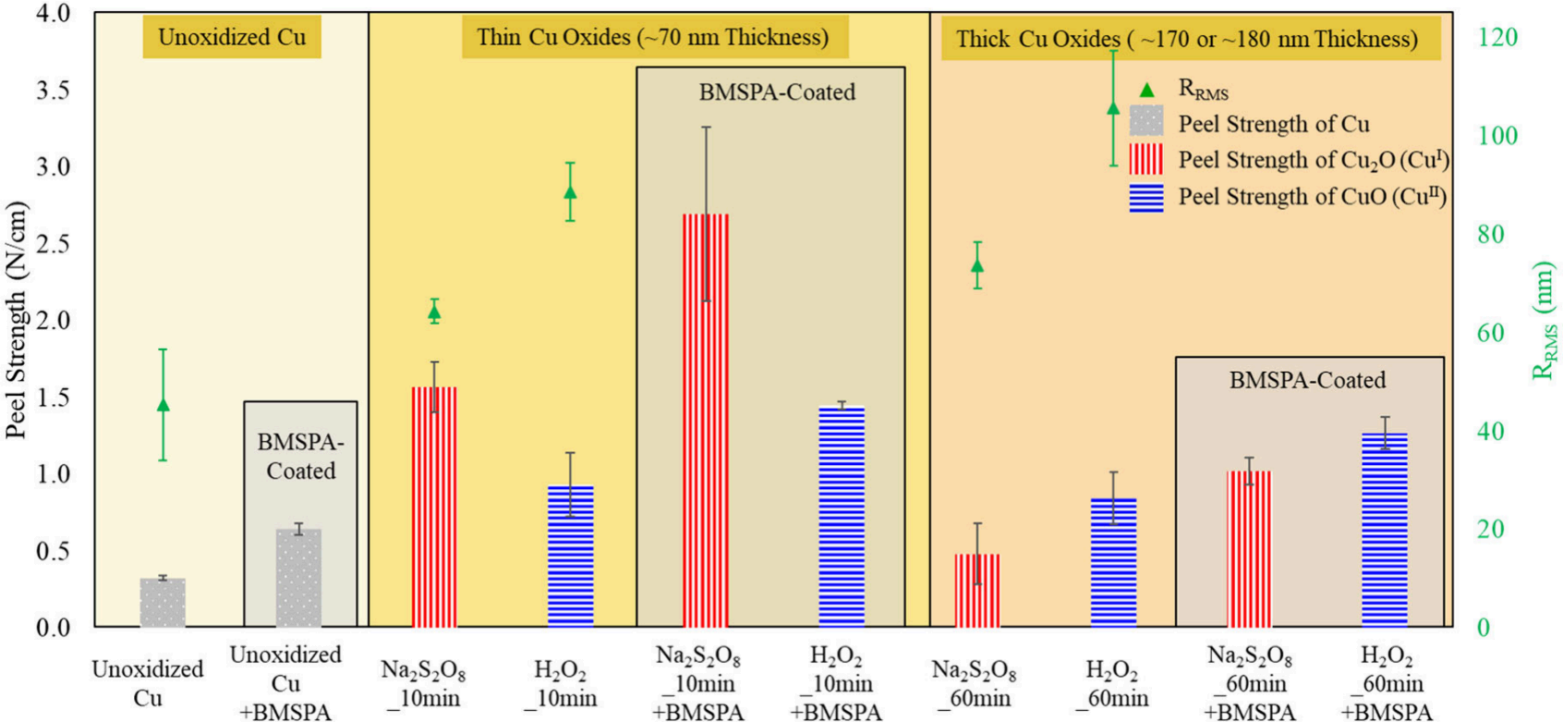
Peel strength of unoxidized Cu, unoxidized and BMSPA-coated Cu (Unoxidized Cu+BMSPA), Cu oxidized for 10 min by Na_2_S_2_O_8_ (Na_2_S_2_O_8__10 min) or H_2_O_2_ (H_2_O_2__10 min), and Cu oxidized for 60 min by Na_2_S_2_O_8_ (Na_2_S_2_O_8__60 min) or H_2_O_2_ (H_2_O_2__60 min), followed with/without BMSPA coating. Error bars are determined by measuring three samples. Peel strength data and error limits for the samples are as follows: Unoxidized Cu: 0.3 N/cm, Unoxidized Cu+BMSPA: 0.6 N/cm, Na_2_S_2_O_8__10 min: 1.6 ± 0.2 N/cm, H_2_O_2__10 min: 0.9 ± 0.2 N/cm, Na_2_S_2_O_8__10 min+BMSPA: 2.7 ± 0.6 N/cm, H_2_O_2__10 min+BMSPA: 1.4 N/cm, Na_2_S_2_O_8__60 min: 0.5 ± 0.2 N/cm, H_2_O_2__60 min: 0.8 ± 0.2 N/cm, Na_2_S_2_O_8__60 min+BMSPA: 1.0 ± 0.1 N/cm, H_2_O_2__60 min+BMSPA: 1.3 ± 0.1 N/cm. Green scatters represent R_RMS_.

AFM analyses presented in Figure 4 were used to quantify the root-mean-square roughness (R_RMS_) of the oxidized Cu foils. Nonoxidized Cu foils present 45 nm R_RMS_. After 60 min of oxidation, the H_2_O_2_ oxidizer increases the R_RMS_ to 106 nm R_RMS_, while Na_2_S_2_O_8_ increases the R_RMS_ to 74 nm R_RMS_. In Figures 4 and S4, the needle-like structure formed by H_2_O_2_ (Figure 3c) generates high R_RMS_ that could promote mechanical interlocking.^28^ Despite the expected improvement in mechanical interlocking, Figure 5 provides contrasting results of the peel test.

### 3.2 Adhesion

The 90° peel tests were performed after the epoxy was cured on the Cu layer (cured at 115 °C for 8 h). Figure 5 shows the peel strengths and the corresponding R_RMS_ as a function of different conditions such as surface oxidation and BMSPA coating. Before the surface oxidation, the peel strength of 0.3 N/cm is comparable to reports in the literature (0.5 ± 0.3 N/cm).^45,46^ After the 10 min-oxidation with Na_2_S_2_O_8_, the peel strength abruptly increases to 1.6 ± 0.2 N/cm; however, the Na_2_S_2_O_8__60 min sample, despite its higher R_RMS_ and expected stronger mechanical interlocking, sharply decreases the peel strength of the Cu-epoxy down to 0.5 ± 0.2 N/cm. This decrease in peel strength of the Na_2_S_2_O_8__60 min sample is associated with stress in the thick layer, which is reported to cause the weakening of bond strength.^47^ This result suggests that thin Cu oxide (∼70 nm, Figure 2b) grown by Na_2_S_2_O_8_ is beneficial for adhesion improvement at the smooth surface. The BMSPA coating increases the peel strength of 10 min-oxidized and 60 min-oxidized Cu with Na_2_S_2_O_8_ to 2.7 ± 0.6 and 1.0 ± 0.1 N/cm, respectively. The effect of BMSPA coating, following Na_2_S_2_O_8_ oxidation, on the peel strength is 2.2 times greater for 10 min-oxidized Cu than 60 min oxidized-Cu. This suggests that BMSPA develops stronger chemical adhesion between the epoxy layer and thin Cu oxide (∼70 nm, Figure 2b) grown by Na_2_S_2_O_8_ at a smooth surface; a discussion is further developed to explain this observation in the next sections (sections 3.3, XPS and AES and 3.4 Raman Spectroscopy).

The oxidation by H_2_O_2_ shows an increase in peel strength to 0.9 ± 0.2 N/cm after 10 min. After 60 min of oxidation, the peel strength decreases to 0.8 ± 0.2 N/cm despite its higher R_RMS_ than the H_2_O_2__10 min sample. Since the peel strength is not associated with interfacial roughness, the peel strength does not demonstrate consistent trends with oxidation time relative to roughness. Thus, for the Cu oxide-epoxy interface with R_RMS_ < 106 nm, chemical adhesion and thickness of Cu oxides are considered to have a more critical impact on overall interfacial adhesion than roughness. The BMSPA coating increases the peel strength of 10 min-oxidized and 60 min-oxidized Cu with H_2_O_2_ by 0.5 N/cm each, which is a more minor effect than the 1.1 N/cm increase observed from the Na_2_S_2_O_8__10 min+BMSPA sample. This result suggests that 10 min Na_2_S_2_O_8_ oxidation is more favorable to developing stronger chemical adhesion at a smooth surface than 10 or 60 min H_2_O_2_ oxidation. The Na_2_S_2_O_8__10 min+BMSPA sample shows the greatest peel strength of 2.7 ± 0.6 N/cm.

### 3.3 XPS and AES

**Figure 6:**
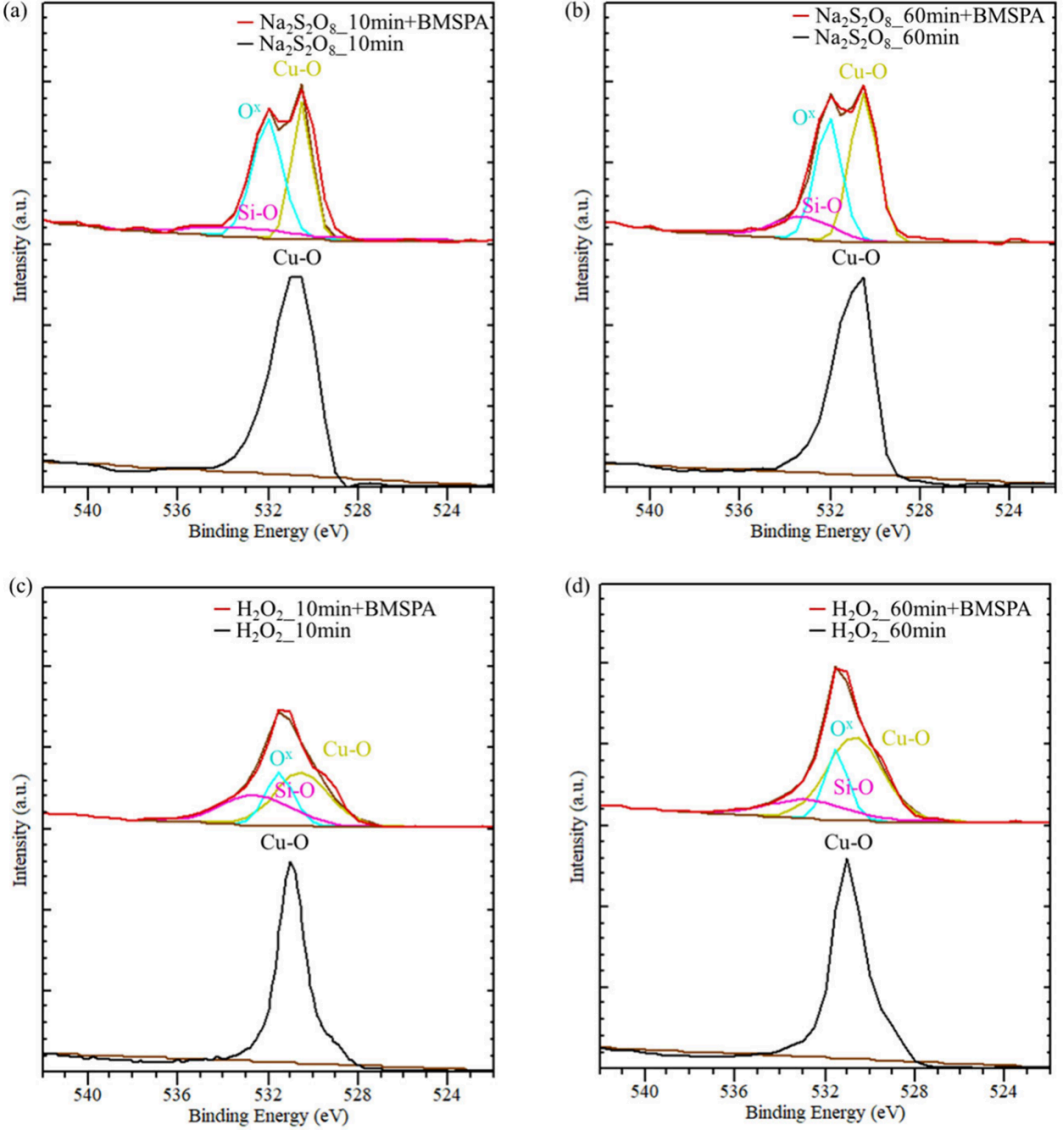
O 1s XPS spectra of Cu oxidized by (a) Na_2_S_2_O_8_ for 10 min (Na_2_S_2_O_8__10 min), (b) Na_2_S_2_O_8_ for 60 min (Na_2_S_2_O_8__60 min), (c) H_2_O_2_ for 10 min (H_2_O_2__10 min), and (d) H_2_O_2_ for 60 min (H_2_O_2__60 min), followed with/without BMSPA coating (red/black line).

**Figure 7:**
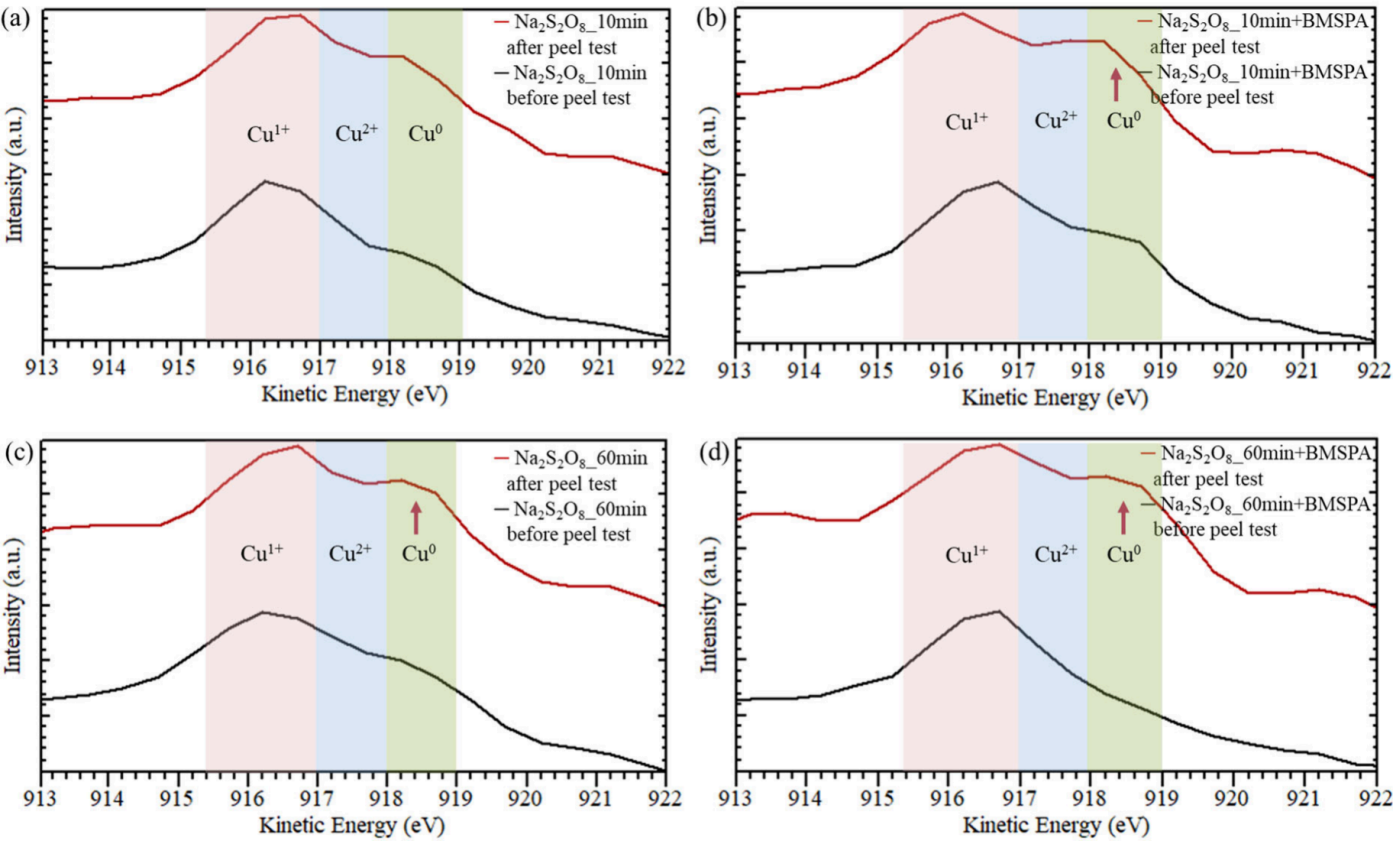
Auger spectra of Cu oxidized by (a) Na_2_S_2_O_8_ for 10 min (Na_2_S_2_O_8__10 min), (b) Na_2_S_2_O_8_ for 10 min, followed by BMSPA coating (Na_2_S_2_O_8__10 min+BMSPA), (c) Na_2_S_2_O_8_ for 60 min (Na_2_S_2_O_8__60 min), and (d) Na_2_S_2_O_8_ for 60 min, followed by BMSPA coating (Na_2_S_2_O_8__60 min+BMSPA) before/after peel test (black/red line). Red arrows indicate peaks appearing after the peel test.

**Figure 8:**
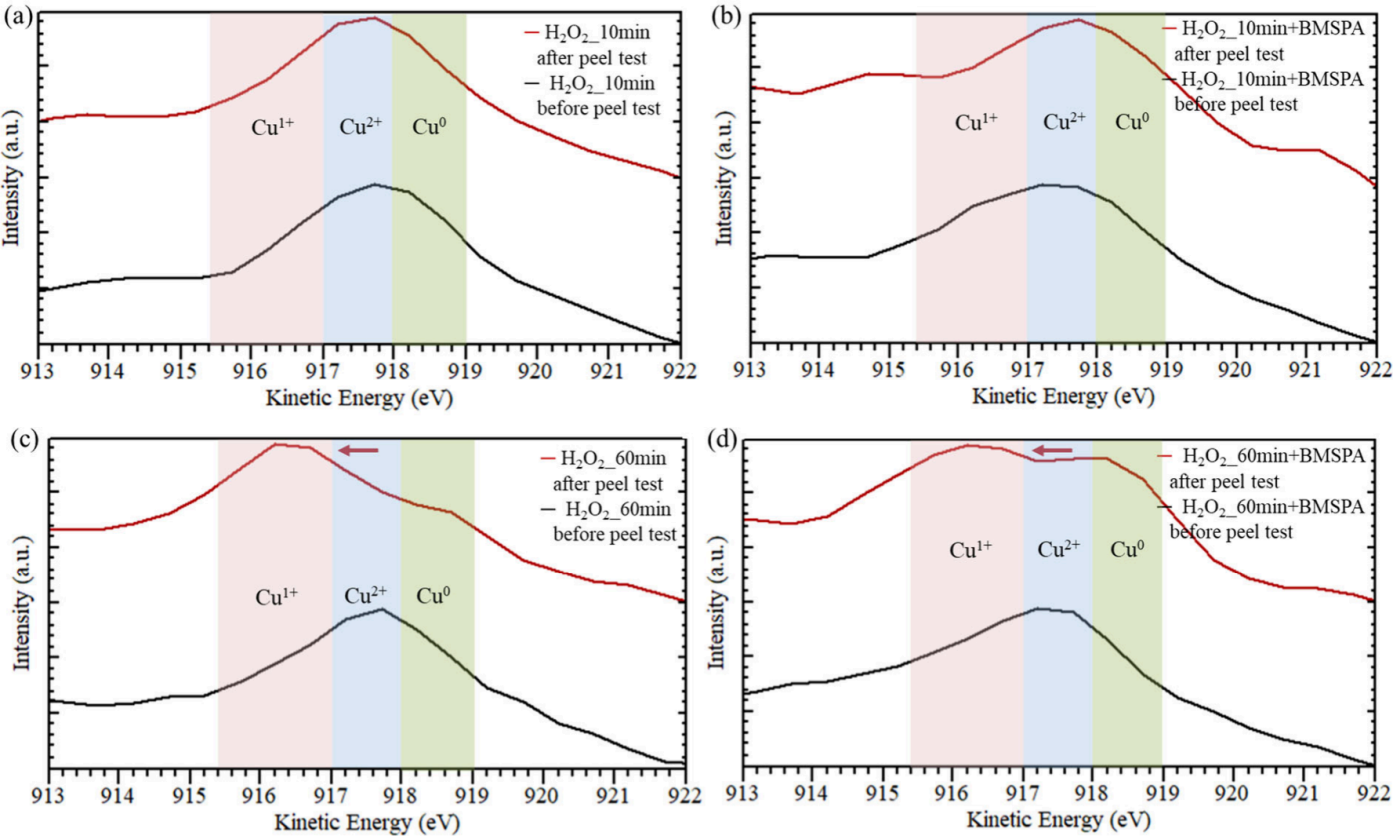
Auger spectra of the Cu surface oxidized by (a) H_2_O_2_ for 10 min (H_2_O_2__10 min), (b) H_2_O_2_ for 10 min, followed by BMSPA coating (H_2_O_2__10 min+BMSPA), (c) H_2_O_2_ for 60 min (H_2_O_2__60 min), and (d) H_2_O_2_ for 60 min, followed by BMSPA coating (H_2_O_2__60 min+BMSPA) before/after peel test (black/red line). Red arrows indicate the dominant peak shifting after the peel test.

XPS and AES results show the elemental composition and chemical interaction associated with adhesion.^48,49^ XPS or AES measurements were carried out on (i) Cu surfaces before molding epoxy (Figures 6–8, S5 and Table 2), (ii) Cu surfaces after the peel test of Cu-molded epoxy (Figures 7, 8), and (iii) epoxy surfaces after the peel test of Cu-molded epoxy (Figures S6,S7).

**Table 2:** Percentage Concentration of Chemical Phases Present in the O 1s Spectra under Various Oxidation Conditions on Cu

	Composition of Phases from the O 1s Spectra (%)		
Sample Name	Cu–O	O^x^ (Cu–O–Si)	Si–O
Na_2_S_2_O_8__10 min+BMSPA	36	50	14
Na_2_S_2_O_8__60 min+BMSPA	46	38	16
H_2_O_2__10 min+BMSPA	44	23	33
H_2_O_2__60 min+BMSPA	59	21	20

Figure 6 shows the O 1s spectra of XPS performed on the grown Cu oxide before and after BMSPA coating. In Figure 6a-d, the peaks at 531 eV associated with Cu oxides (Cu_2_O and CuO) are shown from the Na_2_S_2_O_8__10 min, Na_2_S_2_O_8__60 min, H_2_O_2__10 min, and H_2_O_2__60 min samples.^50^ Cu–O and Si–O are reported to have peaks at 531 and 533 eV, respectively.^50^ We observe that Cu oxidized by Na_2_S_2_O_8_ presents the relatively strong emergence of an O^x^ peak at 532 eV, which is related to the Cu–O–Si formation after the BMSPA deposition in Figure 6a,b;^50^ however, XPS results in Figure 6c,d present that Cu oxidized by H_2_O_2_ shows a weaker O^x^ peak at 532 eV after BMSPA coating, indicating that H_2_O_2_ oxidation is less favorable to forming Cu–O–Si bonds. The hydrolysis of silane produces silanol groups. These silanol groups react with Cu oxides to create Cu–O–Si bonds, which are indicated by the peak at 532 eV in Figure 6a-d. Si–O–Si bonds are also formed from the reaction of silanol groups during the condensation.^51,52^ The percentage concentration of Cu–O–Si in Na_2_S_2_O_8_-oxidized Cu is approximately 2 times greater than that of H_2_O_2_-oxidized Cu, as presented in Table 2. The formation of the Cu–O–Si covalent bonds is also associated with the greatest peel strength of the Na_2_S_2_O_8__10 min+BMSPA sample in Figure 5. In contrast, the weaker Cu–O–Si peaks from the H_2_O_2_-oxidized samples are associated with a relatively low peel strength in Figure 5.

AES was used to assess the major failure interface after the adhesion tests by analyzing the oxidation states of Cu (Cu^0^, Cu^1+^, or Cu^2+^). AES measurements in Figures 7 and 8 were performed on (i) Cu surfaces before molding epoxy and (ii) Cu surfaces after the peel test of Cu-molded epoxy.

Figure 7 shows the AES of Cu oxidized by Na_2_S_2_O_8_. Before molding epoxy and the peel test, the formation of Cu_2_O is validated on the Cu surface by the peak at 916.2 eV associated with Cu^1+^ in Figure 7a-d.^53^ After the peel test of the Cu–epoxy system, we observe a dominant Cu^1+^ peak at 916.2 eV in Figure 7a,^53^ indicating that the Cu_2_O surface is exposed because the Cu_2_O–epoxy interface has poor interfacial adhesion for the Na_2_S_2_O_8__10 min sample. Figure 7b shows the appearance of a Cu^0^ peak at 918.2 eV after the peel test of the Na_2_S_2_O_8__10 min+BMSPA sample,^53^ suggesting that the BMSPA improves adhesion at the Cu_2_O–epoxy interface, thereby shifting the weakest interface to the Cu–Cu_2_O interface. For the Na_2_S_2_O_8__60 min and Na_2_S_2_O_8__60 min+BMSPA samples in Figure 7c,d, C^0^ peaks at 918.2 eV appear after the peel test regardless of the presence of BMSPA.^53^ This suggests that Cu_2_O thickness affects the weakening of the interfacial adhesion, specifically for the interface between Cu and thick Cu_2_O (∼170 nm, Figure 2c).

AES results in Figure 8a,b present that the H_2_O_2__10 min and H_2_O_2__10 min+BMSPA samples exhibit a dominant peak at 917.5 ± 0.3 eV corresponding with Cu^2+^ species before and after the peel test.^54−56^ This AES result indicates that the failure occurs at the CuO–epoxy interface, regardless of whether BMSPA is applied. In Figure 8c,d. The Cu^2+^ peak shifted to the Cu^1+^ peak at 916.2 ± 0.1 eV after the peel test of the H_2_O_2__60 min and H_2_O_2__60 min+BMSPA samples.^53^ The AES results of the H_2_O_2__60 min and H_2_O_2__60 min+BMSPA samples suggest that the failure at the Cu_2_O–CuO interface during the peel test is associated with the CuO thickness, as the H_2_O_2__60 min and H_2_O_2__60 min+BMSPA samples exhibit different failure interface compared to the H_2_O_2__10 min and H_2_O_2__10 min+BMSPA samples. The relatively high stress in the thick layer and the low dislocation mobility of thick Cu oxide cause the brittleness of thick CuO (∼180 nm, Figure 2e) due to high-stress concentration at the interface.^33^

**Figure 9:**
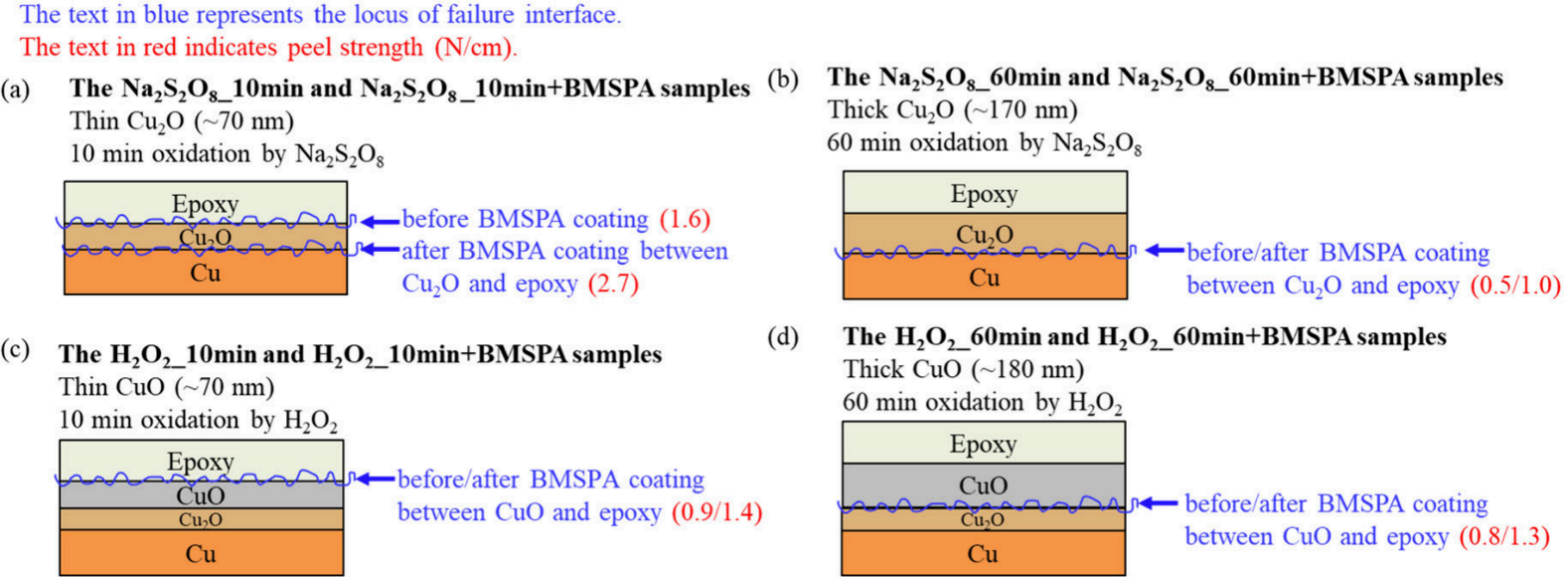
Schematic failure interface of adhesion system of epoxy and Cu oxidized by (a) Na_2_S_2_O_8_ for 10 min (Na_2_S_2_O_8__10 min), (b) Na_2_S_2_O_8_ for 60 min (Na_2_S_2_O_8__60 min), (c) H_2_O_2_ for 10 min (H_2_O_2__10 min), and (d) H_2_O_2_ for 60 min (H_2_O_2__60 min), followed with/without BMSPA coating. The blue and red text represent the locus of the failure interface and peel strength (N/cm), respectively.

In Figure 9a-d and Table S1, the failure mechanism of the Cu-epoxy system is discussed. Cu oxidation mechanism is known as Cu → Cu_2_O → CuO.^57,58^ Thus, in Figure 9c,d, a thin film of Cu_2_O is presumed to be positioned between Cu and CuO before the failure locus analysis.^57−59^

The AES results shown in Figure 7a,b and the peel test shown in Figure 5 present that BMSPA improves adhesion on the relatively thin Cu_2_O layer (∼70 nm, Figure 2b); the Na_2_S_2_O_8__10 min+BMSPA sample shows an ∼70% increase in peel strength compared to the Na_2_S_2_O_8__10 min sample by mitigating failures at Cu_2_O-epoxy interface, as presented in Figure 9a. As shown in Figures 7c,d and 9b, the sample with ∼170 nm-thick Cu_2_O exposes the metallic Cu^0^ interface after the peel test regardless of the BMSPA treatment, indicating that the impact of the BMSPA treatment is less effective. The relatively thick Cu_2_O is thought to increase stress in the thick layer and decrease the dislocation mobility, resulting in high-stress concentration at the interface and brittleness.^33,47,60^

As shown in Figure 9c, the adhesion strength at the CuO–Cu_2_O interface is greater than the CuO–epoxy interface for the H_2_O_2__10 min sample. The effect of BMSPA deposition on ∼70 nm-thin CuO is not verifiable since the adhesion failure mode and peel strength of the CuO–epoxy interface do not present significant changes after the BMSPA deposition, as presented in Figure 9c. Likewise, the BMSPA treatment does not significantly improve the adhesion of relatively thick CuO because this film shows the same adhesion failure mode at the Cu_2_O–CuO interface and similar peel strength for the H_2_O_2__60 min and H_2_O_2__60 min+BMSPA samples, as presented in Figure 9d. The failure at the Cu_2_O–CuO interface is likely due to the brittleness caused by stress in the thick layer and the low dislocation mobility.^33^

### 3.4 Raman Spectroscopy

**Figure 10:**
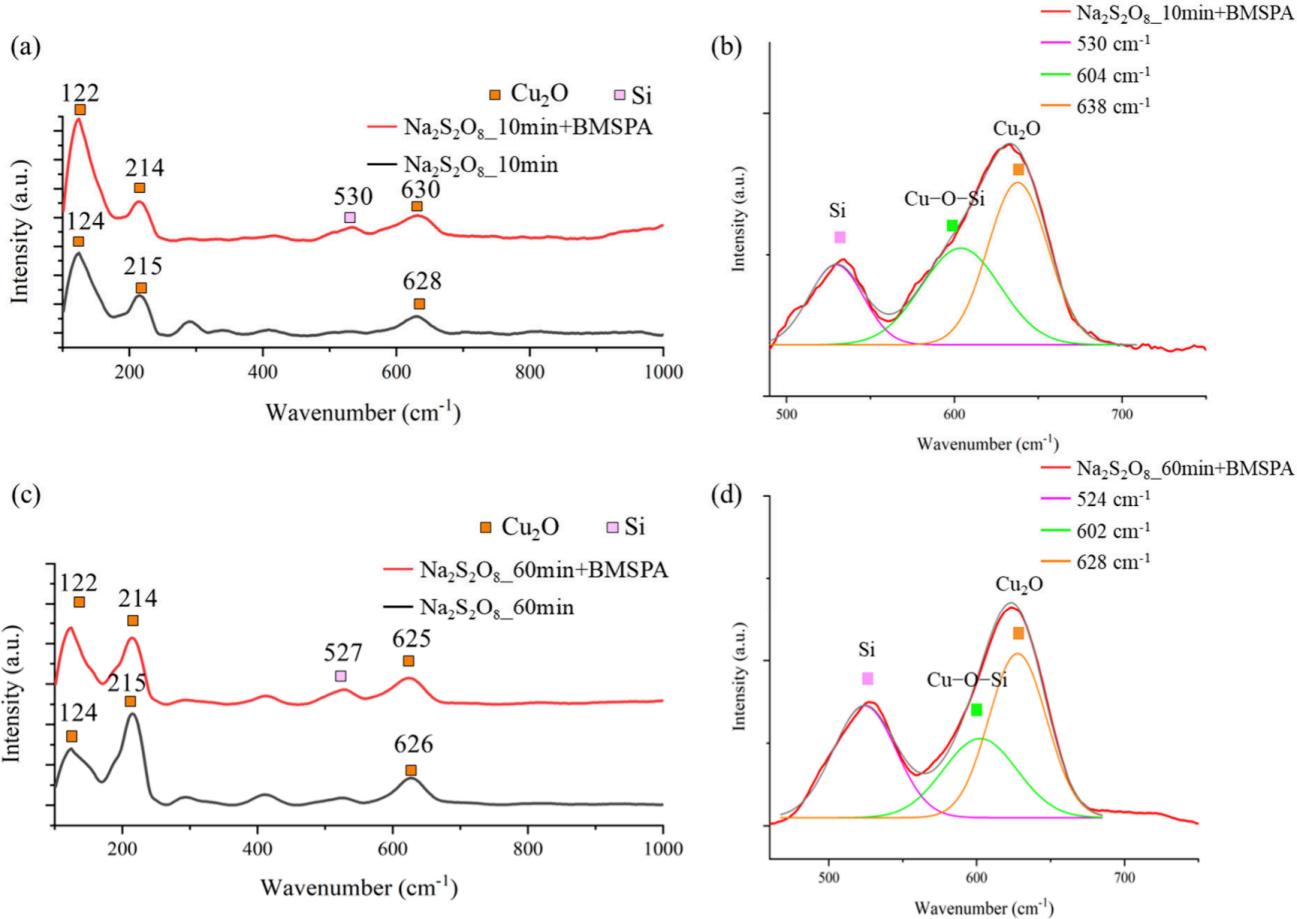
Raman spectra of Cu oxidized by (a) Na_2_S_2_O_8_ for 10 min (Na_2_S_2_O_8__10 min) and (c) Na_2_S_2_O_8_ for 60 min (Na_2_S_2_O_8__60 min), followed with/without BMSPA coating (red/black line). Deconvoluted structures of (a) and (c) are shown in (b) and (d), respectively, within the range of 460 to 690 cm^–1^.

**Figure 11:**
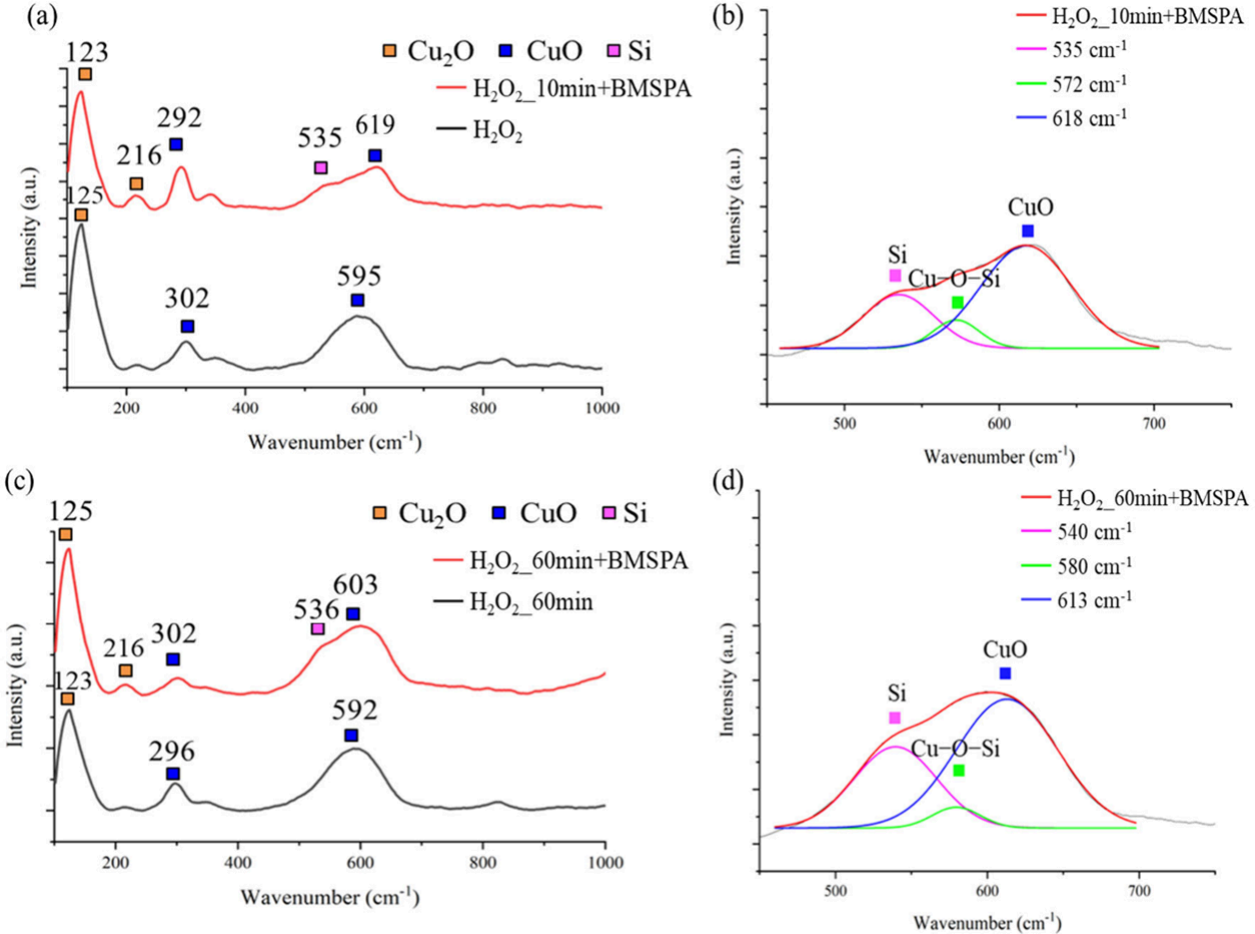
Raman spectra of Cu oxidized by (a) H_2_O_2_ for 10 min (H_2_O_2__10 min) and (c) H_2_O_2_ for 60 min (H_2_O_2__60 min), followed with/without BMSPA coating (red/black line). Deconvoluted structures of (a) and (c) are shown in (b) and (d), respectively, within the range of 460 to 690 cm^–1^.

Raman spectroscopy in Figure 10a-d and Figure 11a-d shows the vibrational modes of the silanized Cu oxide. A Gaussian peak fit is performed to deconvolute the fine structures between 460 and 690 cm^–1^ in Figure 10b,d and Figure 11b,d.^50^

Figure 10 shows the Raman shifts of Cu oxidized by Na_2_S_2_O_8_ before and after BMSPA deposition. The downshift of 24 cm^–1^ of 604 cm^–1^ after the BMSPA silanization, compared with 628 cm^–1^ of Cu_2_O before silanization, is related to Cu–O–Si in Figure 10b.^50^ The downshift is associated with stress after BMSPA coating, which causes intermolecular interaction by introducing the BMSPA to the Cu_2_O system.^61^ Likewise, Figure 10d shows that the Na_2_S_2_O_8__60 min+BMSPA sample with thick Cu_2_O (∼170 nm, Figure 2c) exhibits a downshift of 602 cm^–1^ in comparison with 626 cm^–1^ of Cu_2_O before BMSPA coating, which is related to Cu–O–Si.^50^ In Figure 10b,d, the presence of the peaks at 524 and 530 cm^–1^ is associated with Si–O–Si after BMSPA silanization.^50^Figure S8 additionally shows deposited Si after BMSPA coating by presenting a Si oxide peak at 102 eV.^62^ In Figure 10a-d, the Raman peaks at 122, 124, 214, 215, 626, 628, and 638 cm^–1^ are associated with Cu_2_O.^63^

Figure 11 shows the Raman shifts of Cu oxidized by H_2_O_2_ before and after silanization with BMSPA. In Figure 11b, a peak at 572 cm^–1^ is observed for the H_2_O_2__10 min+BMSPA sample.^64^ The large 23 cm^–1^ downshift of the 572 cm^–1^ peak after BMSPA coating, compared to the value of 595 cm^–1^ of CuO before BMSPA coating, is related to the formation of the Cu–O–Si intermediate phase in Figure 11b. Similarly, the H_2_O_2__60 min+BMSPA sample with thick CuO (∼180 nm, Figure 2e) shows a 12 cm^–1^ downshift of 580 cm^–1^, which is correlated with Cu–O–Si in Figure 11d, compared to CuO peak at 592 cm^–1^ before BMSPA coating.^64^ The downshift is associated with stress, resulting in intermolecular interaction between deposited BMSPA and CuO.^61^ The BMSPA deposition of the H_2_O_2__10 min+BMSPA and H_2_O_2__60 min+BMSPA samples is validated by the Si–O–Si peak at 535 and 540 cm^–1^, respectively.^50^ The Raman shifts detected at 123, 125, and 216 cm^–1^ correspond to Cu_2_O, and the peaks at 292, 296, 302, 592, 595, 613, and 618 cm^–1^ are attributed to CuO.^63^

The Na_2_S_2_O_8__10 min+BMSPA and Na_2_S_2_O_8__60 min+BMSPA samples show more intense Cu–O–Si peaks than the H_2_O_2__10 min+BMSPA and H_2_O_2__60 min+BMSPA samples. In general, Cu_2_O presents higher reactivity compared to Cu and CuO,^65^ which is related to the formation of the Cu–O–Si intermediate phase. The Cu–O–Si bond formation is associated with the greatest peel strength of the Na_2_S_2_O_8__10 min+BMSPA sample in Figure 5. Our Raman spectra with well-defined peak characterization helped develop a fundamental understanding of the impact of Cu_2_O on the Cu–O–Si phase.

## 4 Conclusions

These results help to reveal the behavior of silane adhesion promoters at the interfaces of thin (∼70 nm) Cu_2_O layers and epoxy films. For the first time, we show Raman and XPS evidence of covalent Cu(I)–O–Si bond formation at silane-treated interfaces and a nearly 10-fold increase in adhesion strength. This relationship suggests that relatively smooth Cu interconnects with thin oxides improve the mechanical integrity of Cu-epoxy interfaces; however, relatively thick Cu^I^ layers result in failures at the Cu–oxide interface. Likewise, substrates with Cu^II^ layers typically show facile failures regardless of the CuO thickness or adhesion promoters.

Although thin Cu^I^ layers and “shared oxygen” with adhesion promoters are shown to enhance Cu-epoxy adhesion, Cu^I^ oxides are not thermodynamically stable.^66^ Thus, the long-term stability of the oxide–polymer interface depends on a number of factors that contribute to continued oxidation, and estimating the operating lifetimes of Cu interconnects with epoxy dielectrics is difficult.^29^ The epoxy dielectric is prone to absorb water, and thermal cycling typically leads to plasticization and physical aging.^67^ According to a related study by Brockmann et al., Cu-epoxy integrity degrades via reversible deterioration of the epoxy, irreversible and slow hydration of Cu oxides, and oxygen diffusion through the epoxy, followed by corrosion at the Cu–epoxy interface.^68^ While the covalent bonds afforded by the adhesion promoter are advantageous for improving the initial adhesion strength, their impact on long-term reliability is not well established. This drives the need for modeling, durability studies, and the development of accelerated testing methods such as temperature and humidity stress tests with temperature cycling.

## Supporting Information Available

10 mm wide Cu strip preparation for 90-degree peel test, SEM images of Cu samples, SEM-EDS of Cu samples, 5 μm × 5 μm AFM images of oxidized Cu, Cu 2p XPS spectra of oxidized Cu on Cu side before epoxy molding, Cu 2p XPS spectra on epoxy side after the peel test of Cu-epoxy system, O 1s XPS spectra on epoxy side after the peel test of Cu-epoxy system, Failure interface locus and failure types of various Cu samples, and Si 2p XPS spectra of oxidized Cu before peel test. (PDF)
