# Supplementary material for: Fundamental Insights into Copper-Epoxy Interfaces for High-Frequency Chip-to-Chip Interconnects
Source: ACS Appl Mater Interfaces. 2024 Dec 18;17(1):2480–90. doi: 10.1021/acsami.4c16414 (PMC11784713; doi:10.1021/acsami.4c16414)
Supplement: Supplementary file 1 — am4c16414_si_002.pdf [file am4c16414_si_002.pdf]

Supporting Information

Fundamental Insights into Copper-Epoxy Interfaces for High-Frequency Chip-to-Chip Interconnects

Junghyun Park, Monsuru Dauda*, Mustapha Bello*, Ignace Agbadan*, Anthony Christian Engler*, Jaimal M. Williamson†, Varughese Mathew‡, Sunggook Park§, and John C. Flake**

*Gordon A. and Mary Cain Department of Chemical Engineering, Louisiana State University, Baton Rouge, LA 70803 USA

†Texas Instruments Incorporated, Dallas, TX 75243 USA

‡NXP Semiconductors, Austin, TX 78735 USA

§Department of Mechanical & Industrial Engineering and Center for Bio-Modular Multiscale Systems, Louisiana State University, Baton Rouge, LA 70803 USA

Corresponding Author: johnflake@lsu.edu (John C. Flake)

Total number of pages: 10. Total number of figures: 8. Total number of tables: 1.

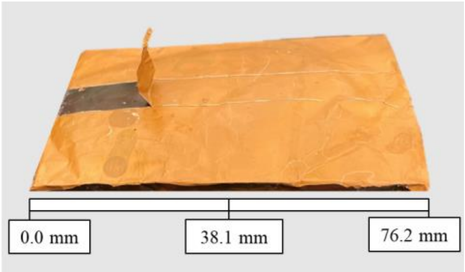

Figure S1. 10 mm wide Cu strip preparation for 90-degree peel test.

Figure S2a-e and Figure S3a-e present SEM-EDS results of Cu under different oxidation conditions. Non-oxidized Cu exhibits indistinguishable bright and dark regions with an O concentration of 0.3 % in both the surface and inner layer, indicating Cu^0. After 10 min of oxidation with $Na_2S_2O_8$ or H_2O_2, ~70 nm thin and brighter region appears, indicating Cu oxides. After 60 min oxidation, the thickness increases to 170 nm for $Na_2S_2O_8$-oxidized Cu and 180 nm for H_2O_2-oxidized Cu. The surface O concentration is 0.9-1.1 % for $Na_2S_2O_8$-oxidized Cu and 1.7-2.0 % for H_2O_2 oxidized Cu. H_2O_2-oxidized Cu shows approximately 2 times greater O concentration than Cu treated with $Na_2S_2O_8$. This difference in O concentration is related to the oxidation state of Cu presented in AES results in Figures 7 and 8, suggesting that $Na_2S_2O_8$ and H_2O_2 primarily produce Cu_2O and CuO, respectively.

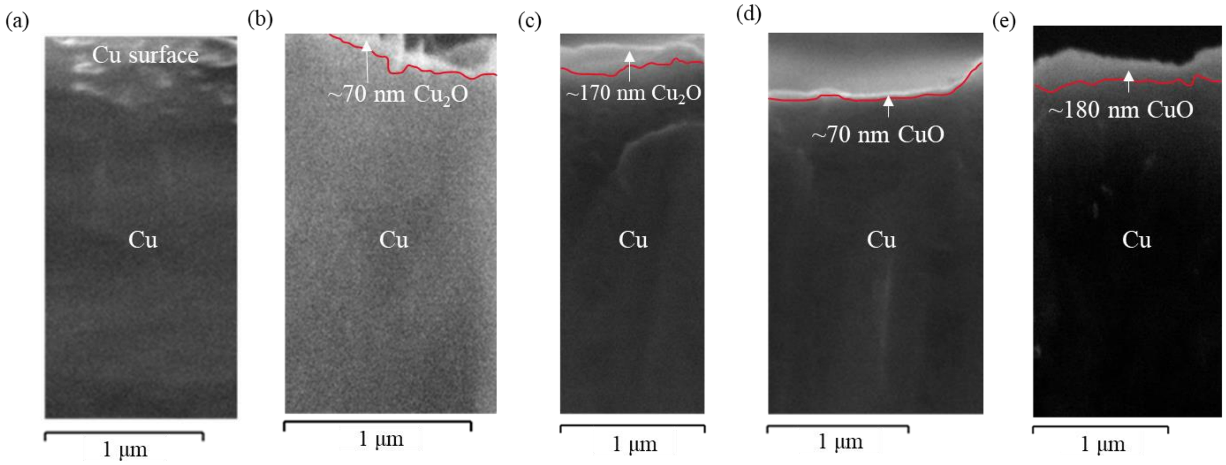

Figure S2. SEM images used for EDS in Figure S3: (a) non-oxidized Cu foil, (b) Cu foil oxidized by $Na_2S_2O_8$ for 10 min ($Na_2S_2O_8$_10min), (c) Cu foil oxidized by $Na_2S_2O_8$ for 60 min ($Na_2S_2O_8$_60min), (d) Cu foil oxidized by H_2O_2 for 10 min (H_2O_2_10min), and (e) Cu foil oxidized by H_2O_2 for 60 min (H_2O_2_60min). Red lines indicate the interfaces between Cu and Cu oxides.

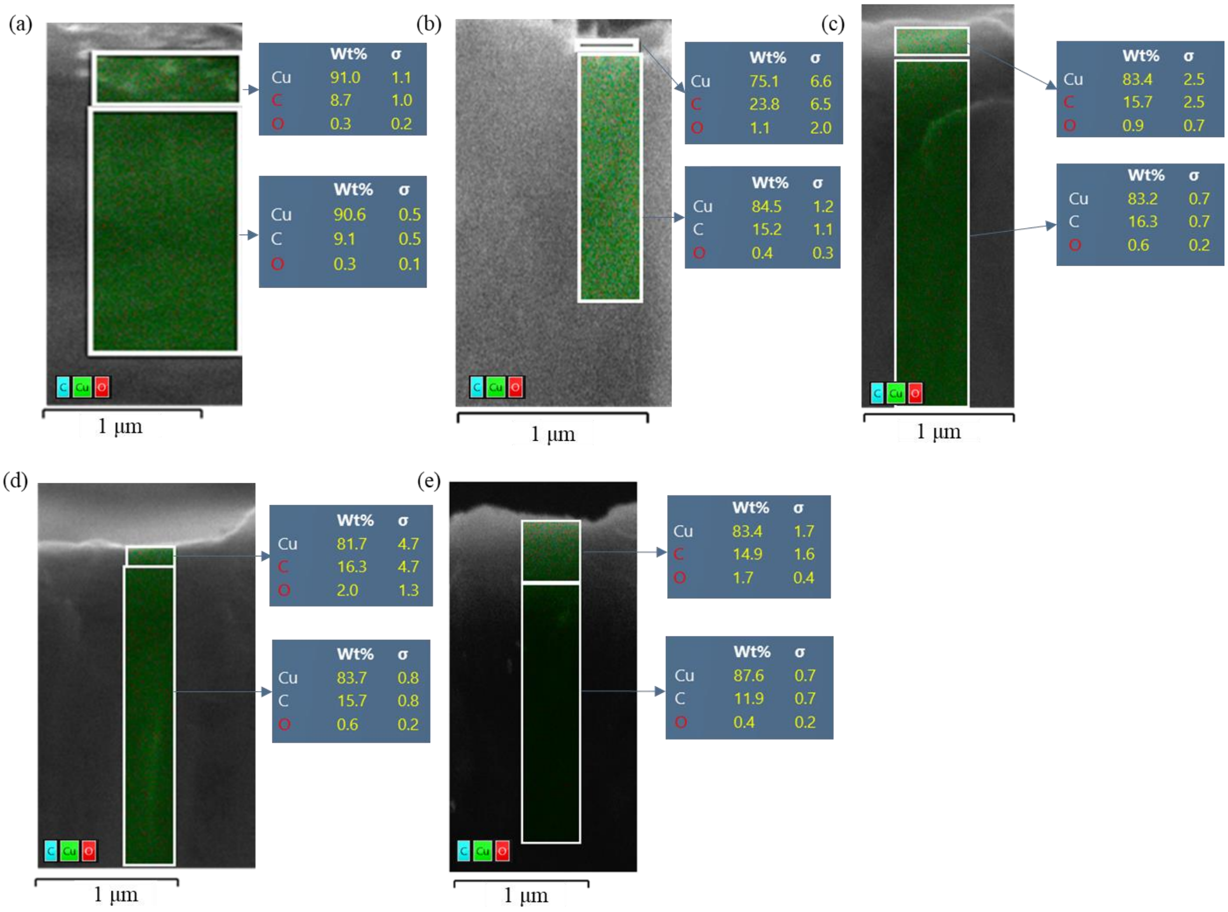

Figure S3. SEM-EDS of (a) non-oxidized Cu foil, (b) Cu foil oxidized by $Na_2S_2O_8$ for 10 min ($Na_2S_2O_8$_10min), (c) Cu foil oxidized by $Na_2S_2O_8$ for 60 min ($Na_2S_2O_8$_60min), (d) Cu foil oxidized by H_2O_2 for 10 min (H_2O_2_10min), and (e) Cu foil oxidized by H_2O_2 for 60 min (H_2O_2_60min).

The surface morphology of Cu oxide is affected by oxidation time, classified as either "peak-valley" or "pore-type" roughness.[1] Peak-valley roughness features relatively regular and shallow

variations, while pore-type roughness is characterized by relatively irregular and deeper pores.[1] As presented in Figure S4a,b, The non-oxidized Cu and Cu oxidized by $Na_2S_2O_8$ for 10 min exhibit peak-valley roughness. The peak-valley roughness is reported to have weaker mechanical adhesion than pore-type roughness due to the limited mechanical interlocking.[1] When the Cu is oxidized by $Na_2S_2O_8$ for 60 min, Figure S4c shows that the surface exhibits deep pores, possibly enhancing the mechanical interlocking. On the other hand, Cu samples oxidized by H_2O_2 for 10 min and 60 min show pore-type roughness, as presented in Figure S4d,e. Cu oxidized by H_2O_2 for 60 min has a coarser and more granular surface, which could enhance the mechanical interlocking.

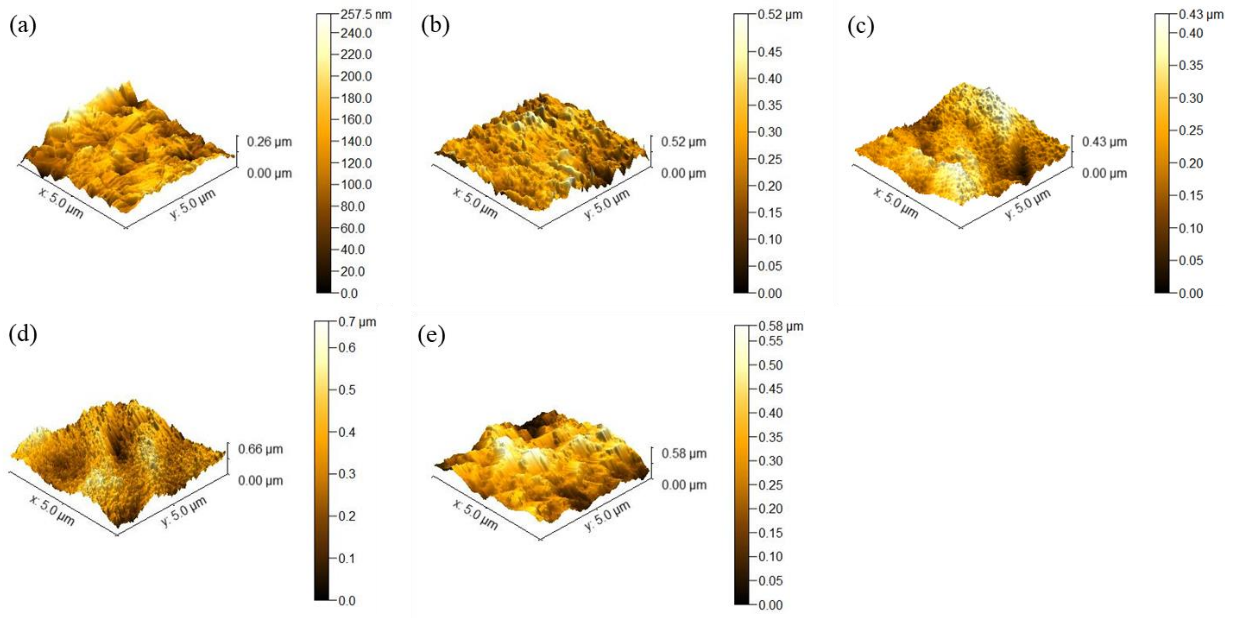

Figure S4. 5 µm x 5 µm AFM images of Cu oxidized (a) for 0 min (45 nm R_{RMS}), (b) by $Na_2S_2O_8$ for 10 min (64 nm R_{RMS}), (c) by $Na_2S_2O_8$ for 60 min (74 nm R_{RMS}), (d) by H_2O_2 for 10 min (89 nm R_{RMS}), and (e) by H_2O_2 for 60 min (106 nm R_{RMS}).

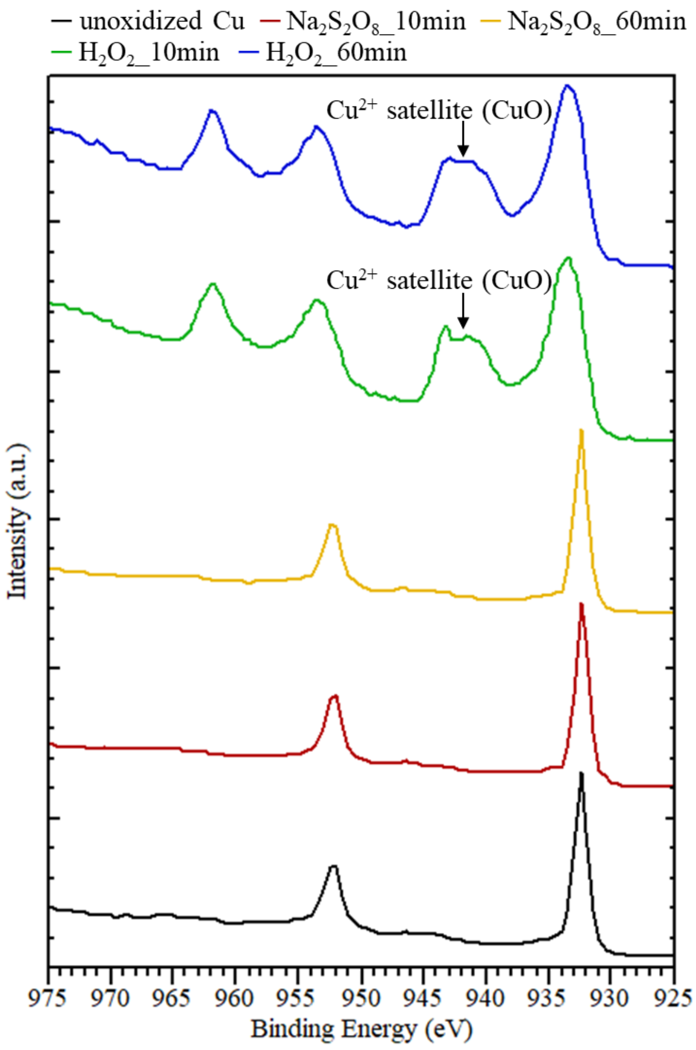

Figure S5. Cu 2p XPS spectra of Cu oxidized for 0 min (black line), by $Na_2S_2O_8$ for 10 min ($Na_2S_2O_8$_10min, red line), by $Na_2S_2O_8$ for 60 min ($Na_2S_2O_8$_60min, yellow line), by H_2O_2 for 10 min (H_2O_2_10min, green line), and by H_2O_2 for 60 min (H_2O_2_60min, blue line).

To determine the failure type (cohesive or adhesive), Cu 2p XPS results on the epoxy surfaces are compared with the AES results on the Cu surfaces after the peel test. On the epoxy surfaces, Cu 2p XPS was performed instead of AES due to the limitation of the AES on the polymer surfaces. The $Na_2S_2O_8$_10min sample shows a cohesive failure by showing the Cu^{1+} peak on the Cu surface in Figure 7a and the Cu^{1+} or Cu^{0} peak at 932-935 eV on the epoxy surface in Figure S6a (c.f. if

there was an adhesive failure at the Cu_2O-epoxy interface, Cu species should not have been detected on the epoxy surface).[2, 3] The epoxy surface of the $Na_2S_2O_8$_10min+BMSPA sample shows the Cu^{1+}/Cu^0 peak after the peel test in Figure S6a. Additional O 1s XPS result of the $Na_2S_2O_8$_10min+BMSPA sample in Figure S7 does not show the Cu-O peak at 531 eV. These Cu 2p and O 1s XPS results on the epoxy surface indicate that the $Na_2S_2O_8$_10min+BMSPA sample has Cu^0 on the epoxy side, and Cu^0 is detected on the Cu side from the AES result in Figure 7b. Hence, the $Na_2S_2O_8$_10min+BMPSPA sample has a cohesive failure at the Cu-Cu_2O interface; however, the $Na_2S_2O_8$_60min and $Na_2S_2O_8$_60min+BMSPA samples have a possibility for both cohesive or adhesive failure since the peaks at 932-935 eV are associated with Cu^{1+} or Cu^0 in Figure S6a. In Figure S7, additional O 1s XPS results on the epoxy surfaces show the Cu-O peaks for the $Na_2S_2O_8$_60min and $Na_2S_2O_8$_60min+BMSPA samples, indicating Cu oxide is on the epoxy side after the peel test; however, the $Na_2S_2O_8$_60min and $Na_2S_2O_8$_60min+BMSPA samples still have a possibility for both cohesive or adhesive failure due to the uncertainty of Cu^0 existence on the epoxy side. The peak at 534 eV is related to the C-O of ester in epoxy, as presented in Figure S7.[4] The H_2O_2_10min and H_2O_2_10min+BMSPA samples show adhesive failure at the CuO-epoxy interfaces after the peel test, exhibiting no Cu species on the epoxy side in Figure S6b. The H_2O_2_60min and H_2O_2_60min+BMSPA samples present cohesive failures at the Cu_2O-CuO interfaces after the peel test by showing the Cu^0/Cu^{1+} peaks and Cu^{2+} peaks at 935-936 eV on the epoxy sides, as presented in Figure S6b and Table S1.

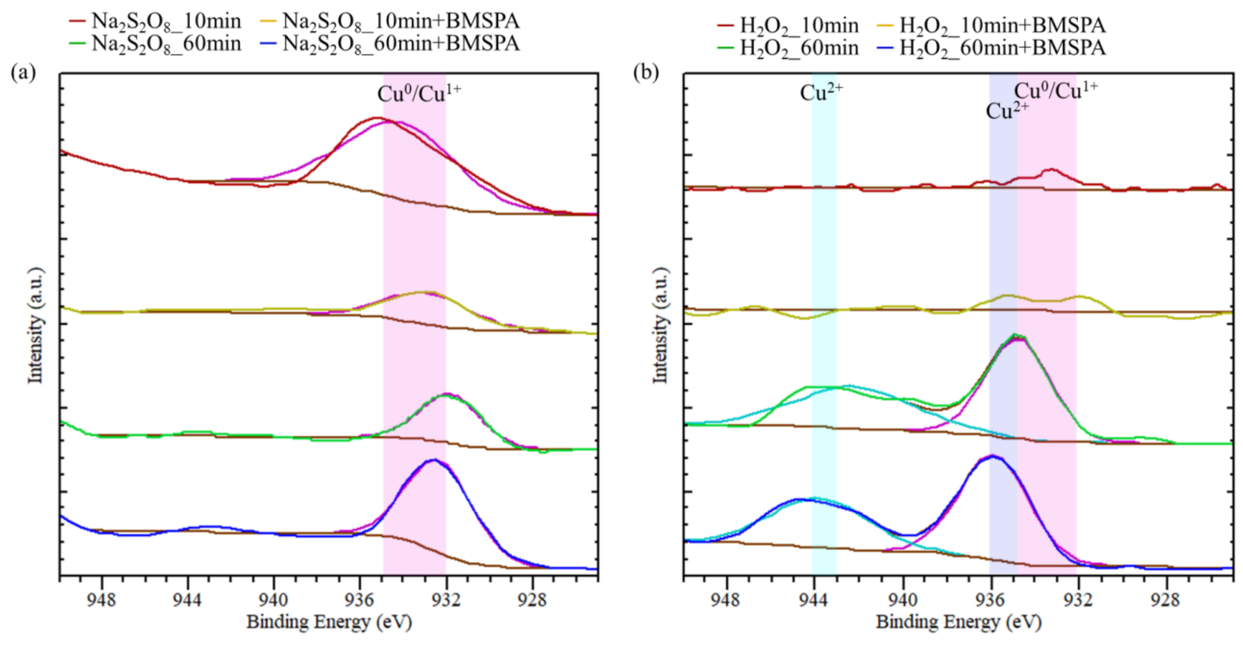

Figure S6. Cu 2p XPS spectra on the epoxy side after the peel test of Cu oxidized (a) by $Na_2S_2O_8$ for 10 min or 60 min and (b) by H_2O_2 for 10 min or 60 min, followed with/without BMSPA coating.

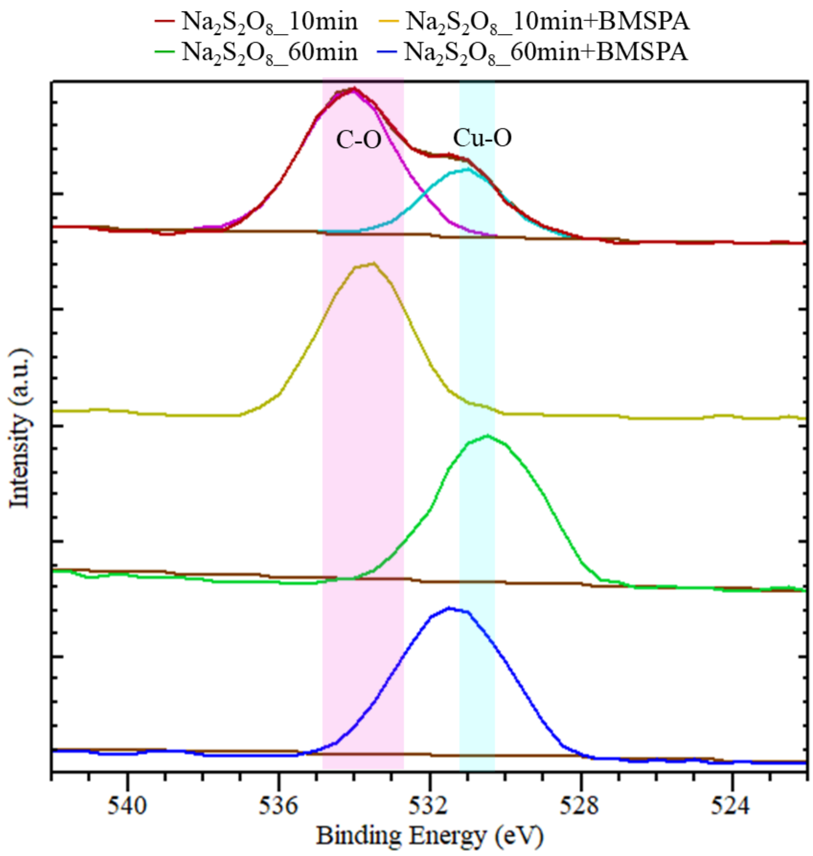

Figure S7. O 1s XPS spectra on the epoxy side after the peel test of Cu oxidized by $Na_2S_2O_8$ for 10 min or 60 min, followed with/without BMSPA coating.

Table S1. Failure interface locus and failure types of various Cu samples

Name	Failure Interface	Failure Type
$Na_2S_2O_8$_10min	Cu_2O-epoxy	Cohesive
$Na_2S_2O_8$_10min+BMSPA	Cu-Cu_2O	Cohesive
$Na_2S_2O_8$_60min	Cu-Cu_2O	Cohesive or Adhesive
$Na_2S_2O_8$_60min+BMSPA	Cu-Cu_2O	Cohesive or Adhesive
H_2O_2_10min	CuO-epoxy	Adhesive

H_2O_2_10min+BMSPA	CuO-epoxy	Adhesive
H_2O_2_60min	Cu_2O-CuO	Cohesive
H_2O_2_60min+BMSPA	Cu_2O-CuO	Cohesive

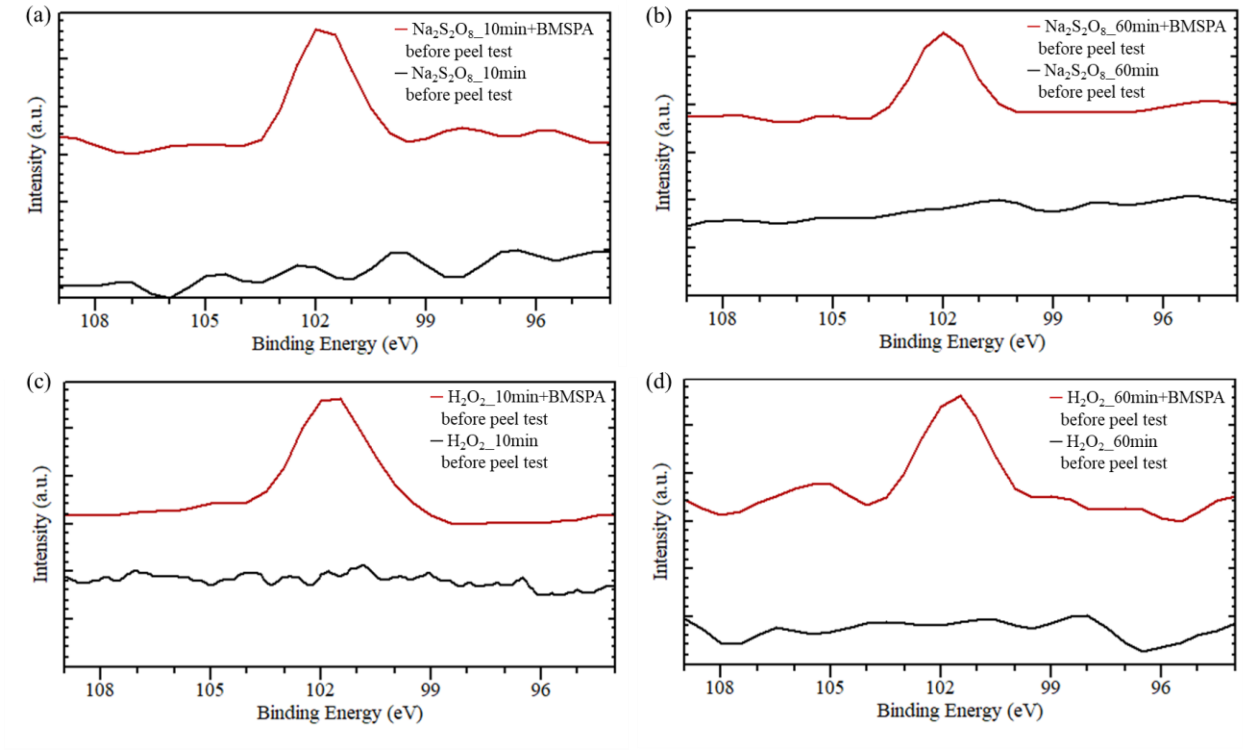

Figure S8. Si 2p XPS spectra of Cu oxidized by (a) $Na_2S_2O_8$ for 10 min ($Na_2S_2O_8$_10min), (b) $Na_2S_2O_8$ for 60 min ($Na_2S_2O_8$_60min), (c) H_2O_2 for 10 min (H_2O_2_10min), and (d) H_2O_2 for 60 min (H_2O_2_60min), followed with/without BMSPA coating (red/black line) before peel test.

REFERENCES

(1) Park, J.; Xu, J.; Engler, A.; Williamson, J.; Mathew, V.; Park, S.; Flake, J. Extending Copper Interconnects and Epoxy Dielectrics to Multi-GHz Frequencies. *IEEE Transactions on Components, Packaging and Manufacturing Technology* **2024**, 1-1. DOI: 10.1109/TCPMT.2024.3399662.
(2) Sreedharan, R.; Mohan, M.; Saini, S.; Roy, A.; Bhattacharjee, K. Intermediate Cu-O-Si Phase in the Cu-SiO2/Si(111) System: Growth, Elemental, and Electrical Studies. *ACS Omega* **2021**, *6* (37), 23826-23836. DOI: 10.1021/acsomega.1c02646.

(3) Bok, S.; Lim, G.-H.; Lim, B. UV/ozone treatment for adhesion improvement of copper/epoxy interface. *Journal of Industrial and Engineering Chemistry* **2017**, *46*, 199-202. DOI: https://doi.org/10.1016/j.jiec.2016.10.031.
(4) López, G. P.; Castner, D. G.; Ratner, B. D. XPS O 1s binding energies for polymers containing hydroxyl, ether, ketone and ester groups. *Surface and interface analysis* **1991**, *17* (5), 267-272.
